# Cultural Adaptation and Validation of the Quality of Dying in Long-Term Care Scale (QoD-LTC) for Spanish Nursing Homes

**DOI:** 10.3390/ijerph18105287

**Published:** 2021-05-16

**Authors:** Daniel Puente-Fernández, Rosel Jimeno-Ucles, Emilio Mota-Romero, Concepción Roldán, Katherine Froggatt, Rafael Montoya-Juárez

**Affiliations:** 1 Doctoral Program of Clinical Medicine and Public Health, University of Granada, 18012 Granada, Spain; 2 Edades Nursing Home, 23160 Jaén, Spain; rju_baeza@hotmail.com; 3 Salvador Caballero Primary Care Centre, Andalusian Health Service, 18012 Granada, Spain; emilio.mota.sspa@juntadeandalucia.es; 4 Department of Statistics and Operational Research, University of Granada, 18071 Granada, Spain; iroldan@ugr.es; 5 Formerly International Observatory on End-of-Life Care, Lancaster University, Lancaster LA1 4YD, UK; kafroggatt@gmail.com; 6 Department of Nursing, University of Granada, 18071 Granada, Spain; rmontoya@ugr.es

**Keywords:** terminal care, nursing homes, long-term care, palliative care, quality of health care, quality indicators

## Abstract

Background: There is a need for instruments that can evaluate the psychosocial quality of dying in nursing homes. The aim of this study was to adapt and validate the Quality of Dying in Long-Term Care scale (QoD-LTC) to the Spanish context. Methods: Descriptive cross-sectional study. Fourteen nurses from 7 facilities in southern Spain assessed 153 residents who died in the centers; validity, reliability, and feasibility were evaluated. Results: The Spanish version consists of 11 items with acceptable reliability (α = 0.681). Three factors model was validated by principal components analysis. A mean of 180.62 (SD = 86.66) seconds is needed to fill it in. An inter-observer 0.753 (95% CI: 0.391–0.900, *p*< 0.001) and intra-observer 0.855 (95% CI: 0.568–0.951 *p* = 0.001) reliability were observed. Weak correlation was observed; positive with mono-item question (0.322) and negative with Eastern Cooperative Oncology Group (ECOG) with a value of (−0.321) and Integrated Palliative outcome scale (IPOS) with a value of (−0.252). Conclusions: The QoD-LTC scale presents an adequate factorial structure, internal consistency, and feasibility to evaluate psychosocial quality of dying in nursing homes. It can be used as a quality indicator.

## 1. Introduction

There is an increasing demand for long-term care services such as nursing homes in Western societies [1,2,3]. This is due to an ageing population and the increasing chronicity and prevalence of multiple diseases [1,2] amongst older people. Nursing homes become the usual place of residence for older people when their care needs become continuous and specialized, beyond the capacity of informal caregivers [2,4]. Therefore, these settings are gaining importance as a place of residence in the last stage of life, given that a significant percentage of older people die in these places [1,5].

Spanish nursing homes offer integral accommodation, either temporary or permanent, to people in a situation of dependence. Their objectives are to achieve a better quality of life and to promote people’s personal autonomy, through the provision of intervention programs and activities that respond to the specific needs of its users [6].

The literature shows that residents at the end of life in nursing homes experience symptoms that worsen their quality of life; Smedbäck et al. (2017), in his retrospective study evaluating the last week of life in Swedish nursing homes, showed that the most frequent symptoms in the last week of life were pain, anxiety, confusion, shortness of breath and nausea [7]. Similar results have been observed for the Spanish context, where interventions that can be considered futile have also been reported [8].

At the end of life, there are several concepts that need to be identified (“quality of care”, “quality of dying” and “quality of death”). Quality of care refers to elements of the environment that occur at death, while quality of dying includes the resident’s symptom burden and other experiences that may be influenced by care and various resident-related factors [9]. Quality of dying is considered to be synonymous with quality of life [9]. Quality of death and dying, on the other hand, is defined as “the degree to which a person’s preferences for dying and the timing of death are consistent with observations of how the person actually died, as reported by others“ [10]. In a recent review, Meier et al. (2016) [10] defined the most representative characteristics of successful dying. Aspects present included “preferences for the dying process” (preparation for death such as advance directives, funeral arrangements), absence of pain, emotional well-being (absence of fear and depression), end of life, treatment preferences, dignity, and family. Since the goals of palliative care include improving the quality of life of patients until death and assisting the family in this process, we can say that palliative care plays a key role in the quality of dying.

Dementia may be another key factor in care provision in these facilities, given that there is a high prevalence in this population [7,8,11,12]. Symptoms and treatments at the end of life are different depending on the presence or absence of dementia [11]. Many tools have been developed to assess the quality of dying and deaths [9,13,14], although none of them have been adapted and validated for the Spanish population and for use in nursing homes. One of the most widely used scales to assess the quality of dying in long-term care facilities is the “Quality of dying in Long-Term Care” (QoD-LTC) [15]. This is a scale validated in the United States, which evaluates psychosocial quality of dying at the last month of life of deceased residents and can be used by professionals and family members alike. One of the advantages is that it takes a short amount of time to complete. It can be completed for deceased patients with or without cognitive impairment [16,17]. Due to the retrospective design of this instrument, it can be used as a quality indicator for care provided by nurses at the end of life.

The validation of a scale in one context does not mean that it will automatically be valid at another time, culture, or context [18]. Therefore, when using a tool in a different culture, it is important to go beyond just direct translation to ensure construct validity and reliability and to reduce the risk of introducing bias into the study [19]. This is especially necessary with such individual and dynamic constructs [20]. A number of relationships are involved in this process between the wishes of the dying person, the ability of others to meet their expectations and the degree of social control exercised over this process, all of which are highly dependent on the culture and context [20]. The end-of-life characteristics and significant changes in residents as they are near death [7,8,21,22,23] make assessment a complex process, with the presence of dementia increasing the difficulties in an assessment [7,24]. This is why it is recommended that instead of using Likert-type measures, the items should be evaluated on a continuous basis, with visual analogue scales (VAS). VAS also allow for greater precision in statistical analysis [25,26,27,28].

The aim of this study was to adapt the QoD-LTC for Spanish nursing homes and evaluate its psycho-metric properties. It is hypothesized that the Spanish version of the QoD-LTC shows an adequate feasibility, reliability, internal consistency, and satisfactory criterion validity. Also, it is hypothesized that QoD-LTC shows a good convergent validity with different scales that assess the quality of palliative care such as the Integrated Palliative Care Outcome Scale (IPOS), Edmonton Symptom Assessment Scale (ESAS) and nurses’ perception of quality of the patient’s death process.

## 2. Materials and Methods

### 2.1. Design

A two-part study was carried out: (1) cultural adaptation using a Delphi method; (2) validation was carried out by conducting a descriptive cross-sectional study in seven nursing homes in the provinces of Granada and Jaén (Spain) following the procedure proposed by Ramada, Serra and Delclós [29]. A cultural adaptation and validation of the QoD-LTC scale was carried out. Data were collected between May 2018 and February 2019.

### 2.2. Sample

For part one, the cultural adaptation used a Delphi procedure. Thirteen experts in the fields of geriatrics, end-of-life or palliative care were contacted. For this study, an expert is understood as any person with extensive clinical, research and/or teaching experience in one of the fields described above.

For part two, two participating nurses in each nursing home with at least 6 months of experience in the center were recruited. They selected and assessed deceased residents who met the following inclusion criteria: (a) Patients who had died within the last 3 months at the nursing home; (b) Who had spent most of the last month of life (at least three weeks) in the nursing home.

All cases who met the above criteria were consecutively sampled and all the informed consents were obtained. No participant loss was reported by nursing home nurses.

This study was undertaken in accordance with basic ethical principles for the responsible conduct of research involving people. The study received the approval of the Research Ethics Committee (1642-N-17), in compliance with Spanish Law (Law 2/2018). Informed consent was sought from participants. All data were anonymized.

### 2.3. Procedure

#### 2.3.1. Cultural Adaptation

For cultural adaptation, the QoD-LTC original scale [15] was translated from English to Spanish and then back to English by two independent bilingual translators. To assess content validity, a synthesis of the translations was evaluated by a committee of experts using the Delphi technique [30]. Experts in geriatrics, end-of-life or palliative care evaluated the relevance, clarity, and levels of meaning and difficulty in response compared to the original language using a Likert-type scale. The agreement was set at 80%.

Once a definitive version was obtained, cognitive interviews were carried out with the first 13 nurses participating in the study. This kind of interview provides information on the way in which individuals process and respond mentally to a questionnaire, to identify factors that may affect the response [31,32]. Cognitive interview records were also used to evaluate feasibility. They were transcribed and read to check that the participants understood the different items.

#### 2.3.2. Validation

A form was developed which included the Spanish version of the QoD-LTC scale and sociodemographic and clinical data. Each of the forms was filled in by the nurses for each of the residents. After measurement, the data were entered into a database for further analysis. To assess convergent validity, the Edmonton Symptom Assessment Scale (ESAS) [33], the Integrated Palliative Outcome Scale (IPOS) [34,35], Eastern Cooperative Oncology Group Scale of Performance Status (ECOG) and Quality of dying during the last month (QoD-LM) were included in the form.

To check reliability among observers, 40% of the cases were randomly selected, and assessments were repeated both by the same nurse (intra-observer reliability) and by another nurse (inter-observer reliability) one week after the first assessment.

### 2.4. Tools

QoD-LTC [15] is a post-death scale designed to evaluate psychosocial quality of dying at the last month of life of institutionalized residents in nursing homes with 11 items, to which professionals or family members who have cared for the deceased respond to the question “How true is it that …?”. In its original version, it has an internal validity of α = 0.66 with three subscales (personhood, closure, and preparatory tasks). Higher mean scores reflect a higher quality of end of life in LTC. The original score is proposed in a Likert-type scale from 1 (not true) to 5 (totally), although for our study it was collected using VAS for reasons previously expressed. Once the questionnaires were obtained, they were measured on a millimetric scale [24,25,26,27]. For correct use, an explanation of how the scale should be answered was included, with a practical example [27].

The following tools were used to assess convergent validity with the QoD-LTC Spanish Scale.

- Edmonton Symptom Assessment System (ESAS) [33]. The ESAS scale has been validated to be filled in by professionals, patients, and caregivers with different diseases, being easily completed and interpreted [35]. ESAS was used regularly in all the nursing homes that participated in the study for symptom assessment. Symptoms have been observed on a conceptual level that may interfere with quality of dying [10].
- Integrated Palliative Outcome Scale (IPOS)[34]: IPOS evaluates palliative care needs in the domains of physical and psychosocial functioning. It is a 17-item scale. Symptoms are assessed on a 0–4 Likert scale. The IPOS was found to be internally consistent, α  =  0.77. Lower scores indicate a better palliative care outcome, the maximum score would be 68. The QoD-LTC scale has been considered to assess the quality of care [9]. In order to assess convergent validity, we have used the IPOS scale.
- Eastern Cooperative Oncology Group Scale of Performance Status (ECOG): it was developed in 1982 and assessing performance status is one such measurement [36]. It describes a patient’s level of functioning in terms of their ability to care for him/herself, daily activity, and physical ability (walking, working, etc.). Functional status has been associated with quality of the dying [16].
- Quality of dying during the last month (QoD-LM): How do you consider the quality of the patient’s death process to have been during his last month? It was evaluated by means of a Likert scale of 1 Terrible, 2 Bad, 3 Normal, 4 Good, 5 Very Good.

### 2.5. Data Analysis

Descriptive analyses were carried out for sociodemographic and clinical data as well as for feasibility.

The Kaiser–Meyer–Olkin (KMO) test and the Bartlett sphericity test were performed to check whether the scale could be subjected to the factorial analysis of principal components carried out later.

Cronbach’s Alpha was calculated for the full scale as well as for each of the subscales. For inter-observer and intra-observer reliability, intra-class correlation coefficient (ICC) was calculated. An ICC of <0.50 indicates poor to fair agreement, ICC = 0.5–0.75 moderate agreement, ICC = 0.75–0.90 good agreement and ICC > 0.90 excellent agreement [37]. Kolmogorov–Smirnov normality tests were performed. Due to the non-normal distribution, non-parametric tests were performed for concurrent validity. Spearman’s rho correlation coefficients between the QoD-LTC score and the results were obtained for the ECOG, ESAS, IPOS, and QoD-LM.

A Spearman’s coefficient of r < 0.39 indicates weak correlation, r = 0.40–0.69 indicates moderate correlation, r > 0.7 indicates strong correlation [38]. *p* < 0.05 was considered significant. IBM SPSS v22.0 [39] was used for data analyses.

## 3. Results

### 3.1. Cultural Adaptation

The expert committee was composed of 13 experts with an average age of 43.3 (32–58) with an average experience of 13 (6–27) years in geriatric, end-of-life or palliative care. An agreement was arrived at for all the items of the scale and the modification of some items was proposed (Table A1 and Table A2). For eight of the items, this consensus was achieved in the second round. For the remaining three items, consensus was achieved in the third round.

A total of 13 cognitive interviews were conducted, in which it was identified that they understood the response mode on the VAS and confirmed the understanding of the modified items. Regarding feasibility, nurses’ average response time for the questionnaire was three minutes (180.62 s), with a standard deviation (SD) of 86.66 s.

### 3.2. Validation

#### 3.2.1. Sample Description

Fourteen nurses from seven centers participated. The mean age was 32.79 (SD = 7.434) years with a mean nursing home experience of 58.86 months and a standard deviation of 48,648.

A total of 153 patients were included. The majority of patients who died in nursing homes were women (56%); the mean age was 86.12 (SD = 11.56) years old at the time of death, widows (60.7%) and most of them died in the facility (57.5%). The most prevalent conditions were dementia (54.9%) and chronic heart disease (39.9%) (Table 1).

#### 3.2.2. Factorial Analysis

The KMO test and Bartlett’s test of sphericity were carried out before a factorial analysis (KMO = 0.653; χ^2^ = 363.090, *p* < 0.001). This scale presented three factors (Table 2): Quality of Care, End-of-life communication, and End-of-life appearance.

#### 3.2.3. Reliability

The QoD-LTC scale showed an adequate reliability (α = 0.681). The α score oscillated among dimensions (Quality of Care α = 0.737; End-of-life communication α = 0.579 and End-of-life Appearance α = 0.575). For each item, statistically significant intra- and inter-class correlation was obtained. Scores correlation ranged from 0.411 to 0.915 for the intra-observer and from 0.371 to 0.837 for the interobserver (Table 3). An inter-observer ICC = 0.753 (95% CI: 0.391–0.900, *p* < 0.001) and an intra-observer ICC = 0.855 (95% CI: 0.568–0.951 *p* = 0.001) was observed for total score scale.

#### 3.2.4. Convergent Validity

A positive correlation was observed between the QoD-LTC scale and its different dimensions with the QoD-LM (r = 0.322) question, and a negative correlation with the IPOS (r = −0.252) and with the ECOG (r = 0.321) scale. It does not correlate with the ESAS scale, although some of its dimensions do (Table 4).

## 4. Discussion

In the present study, we carried out the adaptation and validation of the QoD-LTC scale evaluated by nurses in Spanish nursing homes. The culturally adapted version presented an adequate factorial structure and internal consistency. In addition, it showed good internal reliability, as well as inter- and intra-observer reliability. The adapted version of the QoD-LTC correlates with the IPOS scale and the single-item question and conversely with the ECOG scale. The modified response method is feasible for use in clinical environments and has not affected the reliability of the scale.

During the cultural adaptation process, the expert group changed items that reflected cultural differences between the Spanish and the American context where the instrument was developed. In a Mediterranean culture, family, community ties and the Catholic religion are seen as important factors that influence experiences toward the end of life [40]. Meñaca et al. [40] identified that the Catholic religion with its associated paternalism shapes how family members participate in the entire process of care. This, combined with a resistance to discuss death with older people, means that in many cases residents delegate end-of-life decisions completely to family members [41].

Regarding the item “[RESIDENT] had treatment preferences in writing”, although there is legislation in Spain regulating this practice and advance directives are seen as a positive tool by health professionals, there is evidence that advance directives are not being implemented systematically [42], and a large percentage of the population is unaware of their existence and have never been informed by their doctor about them [43]. The expert group proposed the modification of this item to note the verbal expression of wishes, even if these were not documented, to better reflect cultural practices.

The Spanish version of the QoD-LTC scale showed the same number of factors but a different distribution than the original scale. Our factors are consistent with the qualitative study of the items carried out by Van Soest-Poortvliet et al. (2011) [9], which reported that QoD-LTC primarily assesses the quality of care and the quality of the dying process. Our results coincide with those reported in these studies, where our first factor deals with the quality of care, and the third and fourth items deal with the quality of death.

According to our hypothesis, the adaptation of the QoD-LTC scale presents a similar reliability to the original one (α = 0.681 vs. α = 0.66) [15]. This indicates that the adapted scale and the original scale have the same validity. This is also the case for other scales such as the Palliative Outcome Scale (POS) in its original and Spanish version for patients [44,45]. All items showed a statistically significant degree of agreement between different evaluators in assessing the same case with a different grade of agreement. In the same way, the test–retest shows a statistically significant result in all evaluated scores.

The negative correlation observed with the IPOS scale reinforces that QoD-LTC evaluates a construct which is similar to the quality of palliative care, having among its components aspects that have been identified as potentially forming part of this construct [9]. Although the weak negative correlation indicates that they are not the same construct. Conversely, the better the scores on the QoD-LTC scale, the better the quality of palliative care.

The non-correlation observed with the ESAS scale shows that psychosocial quality of dying is not related to symptom intensity, as has been observed in other studies [46].

In terms of functionality, it is observed that the worse the functionality, the worse the quality of dying. Low correlation indicates that it is not the same construct. Finally, it has been observed that the general assessment of QoD-LM correlates positively with the results of our scale.

The QoD-LTC scale evaluates psychosocial quality of dying. This construct is different if it is from the perspective of the professional, the family member or the patient [10]. As they are different populations, these changes could affect the psychometric characteristics of the scale, so an individual validation for each population is necessary. Nevertheless, the literature is clear in demonstrating that the QoD-LTC is a useful tool [9,14,16].

The availability of this revised instrument when adapted will allow researchers in the Spanish context to undertake comparisons with other contexts [16], thus facilitating the extrapolation of data and the development and comparison of intervention programs at the end of life. This approach can be complemented by qualitative analysis and generate mixed-method studies that provide an integrative approach to the clinical reality in this regard, as well as evaluate end-of-life interventions.

It must be pointed out as a limitation that the nursing home facilities are usually private (71.53%) [43], so their participation in research studies is voluntary. Similarly, it was not possible to select the original cases randomly, but rather, these were chosen according to whether the patients died within the data collection period (consecutively). Although cases were not randomly selected, the characteristics of the sample coincide with those of other studies in the Spanish population [8,11,47,48]. Despite having been evaluated by the expert committee, it would be interesting to test the acceptability and practicability of the scale in nursing homes.

As regards future lines of research, it would be recommended to carry out the adaptation and validation of this instrument into Spanish for its completion by family members, as well as to compare the views of professionals with those of family members, which are different^41^. It would also be interesting to see if this instrument can be filled in by other care home professionals such as physicians, psychologists or social workers. There is further research to be undertaken with specific variables such as underlying health conditions, or the way death occurs, on the QoD-LTC score.

## 5. Conclusions

Validity, reliability, and feasibility of the Spanish version of the QoD-LTC scale have been evaluated, showing psychometric properties comparable to the original version. QoD-LTC is a useful scale applicable to the Spanish-speaking context to assess psychosocial quality of dying.

QoD-LTC is an instrument administered and interpreted by nursing professionals that can facilitate resource and care management. It can also guide the evaluation of protocols that aim for a good quality of dying in nursing homes. This instrument can be an indicator of the quality of the end-of-life processes in these centers.

## Figures and Tables

**Table 1:** Sociodemographic and clinical characteristics of the residents.

Variables	Total Sample *n* = 153	
Age, M (SD)	87.62	(11.56)
Female, *n* (%)	91	(59.5)
Marital status widower, *n* (%)	92	(60.7)
Place of death		
Home, *n* (%)	1	(7)
Hospital, *n* (%)	64	(41.8)
Nursing-Home, *n* (%)	88	(57.5)
Coexisting conditions		
Oncologic disease, *n* (%)	26	(17)
Chronic lung disease	22	(14.4)
Chronic heart disease	61	(39.9)
Dementia	84	(54.9)
Vascular neurological disease	31	(20.3)
Degenerative neurological disease	21	(13.7)
Chronic liver disease	2	(1.3)
Chronic kidney failure	19	(12.4)

**Table 2:** Factorial load of the QoD-LTC scale adapted to the Spanish context.

Items	Factor 1. Quality of Care	Factor 2. End-of-Life Communication by the Resident	Factor 3. End-of-Life Appearance
[RESIDENT] received compassionate physical touch daily.	**0.593**	−0.154	0.351
[RESIDENT’s] dignity was maintained.	**0.730**	0.074	0.136
[RESIDENT’s] physician knew [HIM/HER] as a whole person.	**0.848**	−0.177	0.096
There was a nurse or aide with whom [RESIDENT] felt comfortable.	**0.737**	0.136	0.020
Someone was designated to make decisions in their place [Resident] in the case that they could no longer do so.	**0.647**	0.100	0.082
[RESIDENT] indicated [HE/SHE] was prepared to die.	−0.182	**0.728**	0.178
[Resident] communicated their preferences with respect to treatment.	0.094	**0.820**	0.085
[Resident] expressed how they wanted their funeral and/or other matters concerning their body to be after their death.	0.258	**0.638**	−0.419
[RESIDENT] was kept clean.	0.362	−0.094	**0.410**
[RESIDENT] was able to retain [HIS/HER] sense of humor.	0.085	0.333	**0.763**
[HE/SHE] appeared to be at peace.	0.316	0.113	**0.785**

The extraction was based on Principal Component Analysis using the Varimax rotation method with Kaiser normalization. Bold font indicates the factor loading of the item on the factor to which it was assigned.

**Table 3:** Results of intra- and inter-observer variability.

Items	Intra-Observer (*n* = 46)		Inter-Observer (*n* = 65)	
	ICC	*p*	ICC	*p*
[RESIDENT] was kept clean.	0.588 **	0.002	0.371 *	0.033
[RESIDENT] received compassionate physical touch daily.	0.755 ***	0.000	0.637 **	0.000
[RESIDENT’s] dignity was maintained.	0.672 **	0.000	0.404 *	0.022
[RESIDENT’s] physician knew [HIM/HER] as a whole person.	0.714 **	0.000	0.548 *	0.001
There was a nurse or aide with whom [RESIDENT] felt comfortable.	0.440 *	0.027	0.741 **	0.000
[RESIDENT] was able to retain [HIS/HER] sense of humor.	0.615 **	0.000	0.773 ***	0.000
[RESIDENT] indicated [HE/SHE] was prepared to die.	0.758 ***	0.000	0.660 **	0.000
[HE/SHE] appeared to be at peace.	0.411 *	0.040	0.421 *	0.015
[Resident] communicated their preferences with respect to treatment.	0.726 **	0.000	0.823 ***	0.000
Someone was designated to make decisions in their place [Resident] in the case that they could no longer do so.	0.633 **	0.026	0.837 ***	0.000
[Resident] expressed how they wanted their funeral and/or other matters concerning their body to be after their death.	0.915 ****	0.000	0.787 ***	0.001

ICC: Intraclass Correlation Coefficient. * poor agreement, ** moderate agreement, *** good agreement, **** excellent agreement.

**Table 4:** Convergent validity of the QoD-LTC scale.

Scales		ECOG		ESAS		IPOS		Mono-Item	
		r	*p*	r	*p*	r	*p*	r	*p*
QoD-LTC	Total score	−0.321 *	0.000	−0.153	0.067	−0.252 *	0.002	0.322 *	0.000
	Factor 1	−0.157	0.120	−0.237 *	0.004	−0.303 *	0.000	0.210*	0.010
	Factor 2	−0.190 *	0.019	0.272 *	0.001	0.232 *	0.005	0.235 *	0.004
	Factor 3	−0.292 *	0.000	−0.198 *	0.017	−0.298 *	0.000	0.270 *	0.001

ECOG: Eastern Cooperative Oncology Group Scale of Performance Status; ESAS: Edmonton Symptom Assessment System; IPOS: Integrated Palliative Outcome Scale. Mono-item: Quality of dying during the last month; QoD-LTC: Quality of Dying in Long-Term Care. r = Spearman’s rho; * = weak correlation. *p* = *p* value.

## Data Availability

The data presented in this study are available upon request from the corresponding author. The data are not publicly available due to privacy restrictions

## Appendix A

**Table A1:** Changes proposed by the experts for the items of the QoD-LTC scale.

Original	Adaptation.
[RESIDENT] had treatment preferences in writing.	[Resident] communicated their preferences with respect to treatment.
[RESIDENT] had named a decision-maker in the event that [HE/SHE] was no longer able to make decisions.	Someone was designated to make decisions in their place [Resident] in the case that they could no longer do so.
[RESIDENT] had funeral arrangements planned.	[Resident] expressed how they wanted their funeral and/or other matters concerning their body to be after their death

## Appendix B

**Table A2:** ESCALA -QoD-LTC.

Ítem ¿Cómo de Cierto es Que (el Paciente)…? *Item How True Is It That (the Patient)…*		Para Nada/*Not at All*							Completamente/*Completely*			

1	Se mantuvo aseado.											
										
	*[RESIDENT] was kept clean.*											
2	Se le proporcionó un contacto físico cariñoso todos los días.											
									
	*[RESIDENT] received compassionate physical touch daily.*											
3	Se preservó su dignidad.											
									
	*[RESIDENT’s] dignity was maintained.*											
4	Su médico lo trato de forma integral, atendiendo a todas sus facetas.											
									
	*[RESIDENT’s] physician knew [HIM/HER] as a whole person.*											
5	Tenía un enfermero o auxiliar con quien se sentía cómodo.											
									
	*There was a nurse or aide with whom [RESIDENT] felt comfortable.*											
6	Mantuvo el sentido del humor que lo caracterizaba.											
									
	*[RESIDENT] was able to retain [HIS/HER] sense of humor. [RESIDENT]*											
7	Expresó que estaba preparado para morir.											
									
	*[RESIDENT] indicated [HE/SHE] was prepared to die.*											
8	Parecía estar en paz.											
									
	*[HE/SHE] appeared to be at peace.* *[RESIDENT]*											
9	Comunicó sus preferencias con respecto al tratamiento.											
									
	[*Resident] communicated their preferences with respect to treatment.*											
10	Se nombró a alguien que tomara las decisiones en su lugar en caso de que ya no pudiera.											
									
	*Someone was designated to make decisions in their place [Resident] in the case that they could no longer do so.*											
11	Expresó como quería que fuera su funeral y/o otras cuestiones relativas a su cuerpo después de su fallecimiento.											
									
	*[Resident] expressed how they wanted their funeral and/or other matters concerning their body to be after their death.*											

The background colour of the table is where the participants should mark the point where the answer is located.
